# Identification of autophagic target RAB13 with small‐molecule inhibitor in low‐grade glioma via integrated multi‐omics approaches coupled with virtual screening of traditional Chinese medicine databases

**DOI:** 10.1111/cpr.13135

**Published:** 2021-10-10

**Authors:** Wei Su, Minru Liao, Huidan Tan, Yanmei Chen, Rongyan Zhao, Wenke Jin, Shiou Zhu, Yiwen Zhang, Li He, Bo Liu

**Affiliations:** ^1^ Department of Neurology and State Key Laboratory of Biotherapy and Cancer Center West China Hospital Sichuan University Chengdu China; ^2^ State Key Laboratory of Biotherapy and Cancer Center West China Hospital Sichuan University Collaborative Innovation Center of Biotherapy Chengdu China

**Keywords:** autophagy, cancer therapy, gallic acid, Low‐grade gliomas (LGG), RAB13, small‐molecule inhibitor, Traditional Chinese Medicine (TCM), ZFP36L2

## Abstract

**Objectives:**

Autophagy, a highly conserved lysosomal degradation process in eukaryotic cells, has been widely reported closely related to the progression of many types of human cancers, including LGG; however, the intricate relationship between autophagy and LGG remains to be clarified.

**Materials and methods:**

Multi‐omics methods were used to integrate omics data to determine potential autophagy regulators in LGG. The expression of ZFP36L2 and RAB13 in SW1088 cells was experimentally manipulated using cDNAs and small interfering RNAs (siRNA). RT‐qPCR detects RNAi gene knockout and cDNA overexpression efficiency. The expression levels of proteins in SW1088 cells were evaluated using Western blot analysis and immunofluorescence analysis. Homology modelling and molecular docking were used to identify compounds from Multi‐Traditional Chinese Medicine (TCM) Databases. The apoptosis ratios were determined by flow cytometry analysis of Annexin‐V/PI double staining. We detect the number of autophagosomes by GFP‐MRFP‐LC3 plasmid transfection to verify the process of autophagy flow.

**Results:**

We integrated various omics data from LGG, including EXP, MET and CNA data, with the SNF method and the LASSO algorithm, and identified ZFP36L2 and RAB13 as positive regulators of autophagy, which are closely related to the core autophagy regulators. Both transcription level and protein expression level of the four autophagy regulators, including ULK1, FIP200, ATG16L1 and ATG2B, and LC3 puncta were increased by ZFP36L2 and RAB13 overexpression. In addition, RAB13 participates in autophagy through ATG2B, FIP200, ULK1, ATG16L1 and Beclin‐1. Finally, we screened multi‐TCM databases and identified gallic acid as a novel potential RAB13 inhibitor, which was confirmed to negatively regulate autophagy as well as to induce cell death in SW1088 cells.

**Conclusion:**

Our study identified the key autophagic regulators ZFP36L2 and Rab13 in LGG progression, and demonstrated that gallic acid is a small molecular inhibitor of RAB13, which negatively regulates autophagy and provides a possible small molecular medicine for the subsequent treatment of LGG.

## Graphical abstract

The two positive autophagy regulators ZFP36L2 and RAB13 in Low‐grade Glioma patients were discovered by SNF method and Lasso method, and it was demonstrated that gallic acid can negatively regulate autophagy by inhibiting RAB13 for future LGG therapeutics.

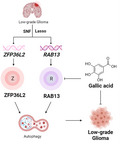

## 1 INTRODUCTION

Gliomas, which are among the cancers that exhibit the poorest prognosis, have been widely reported to be the most common primary central nervous system (CNS) tumours. Low‐grade glioma (LGG) includes WHO grade II diffuse astrocytic and oligodendroglial tumours.^1^, ^2^ A kinase‐interacting protein 1 (AKIP1) has been reported overexpressed in LGG samples and it regulates NF‐κB and AKT pathway through the modulation of CXCL1 and CXCL8, leading to increased proliferation and invasion and decreased apoptosis of U‐87 and U‐252 MG cell.^3^ Moreover, apoptosis evasion phenotype is frequently observed in LGG, including the decrease of BH3‐only proteins PUMA, Bad and Bim.^4^ Although some studies have indicated possible mechanisms of LGG pathogenesis, more efforts are in need to clarify the abnormal alterations in LGG and find new therapeutic strategy.

Autophagy, a regulated mechanism of the cell that removes unnecessary or dysfunctional components, has been demonstrated to play the dual functions in tumour biology: autophagic activation promotes cancer cells survival or contributes to cancer cell death.^5^, ^6^, ^7^, ^8^ Autophagy‐associated signalling pathways, including the ERK1/2 pathway, PI3K/AKT/ mTOR pathway and NF‐κB pathway, act as tumour suppressors or protect tumour cells against chemotherapy/radiotherapy‐induced cytotoxicity in gliomagenesis.^6^ Recently, accumulating evidence has revealed that autophagy‐mediated pro‐survival signals drive the resistance to anti‐angiogenic chemotherapies in glioblastomas.^9^, ^10^ In addition, the autophagic proteins LC3, Beclin‐1 and p62 have been also found less expressed in LGG than in high‐grade glioma (HGG) and the higher levels of LC3 and p62 are closely associated with poor prognosis.^11^ Moreover, recent studies have indicated that inhibition of autophagy or knockdown of autophagy gene expression may serve as a strategy to increase the cytotoxicity of TMZ in LGG therapies.^6^ Since protective autophagy is closely associated with glioma, it might be beneficial to identify critical autophagic regulators in LGG to provide potential druggable targets for potential LGG therapy. Although several therapeutic interventions, such as surgery, radiotherapy and chemotherapy, have been applied to treat LGG, severe side effects of these therapeutics and lack of targeted drugs for LGG contribute to the high mortality of LGG. Thus, developing new strategies for candidate small‐molecule drugs should be necessary to improve potential LGG therapy.^12^, ^13^

Of note, Traditional Chinese Medicine (TCM) has been regarded as new sources of candidate small‐molecule drugs since natural products have highly diverse bioactivities.^14^, ^15^ More recently, some studies have been performed to investigate the effect of TCM extracts or compounds on autophagy for cancer therapeutic purposes.^16^, ^17^ For example, curcumin is a widely recognized as a TCM inducer of autophagy‐dependent death, which was reported to inhibit gliomas in vitro and in vivo via Akt/mTOR/P70S6K pathway and ERK1/2 pathway.^18^, ^19^ Additionally, TCM celastrol suppresses the viability of human U251, U87‐MG and C6 glioma cells by modulating ROS/JNK and Akt/mTOR signalling pathways, indicating the potentially therapeutic effect of TCM celastrol on gliomas.^20^

In this study, we applied multi‐omics approaches to integrate omics data and identify potential autophagic regulators in LGG. As a result, two autophagic regulators, ZFP36L2 and RAB13 were identified in the regulatory network of LGG. Subsequently, we demonstrated that they are positive modulators of autophagy, while the new autophagic regulator RAB13 was upregulated in LGG and the higher expression is closely associated with poor prognosis. Moreover, based upon multi‐TCM Databases, we identified gallic acid as an RAB13 inhibitor, which suppressed the proliferation of SW1088 cells by reducing autophagy. Altogether, positive regulator RAB13 may be a promising druggable target and gallic acid is a potential candidate drug in the treatment of LGG.

## 2 MATERIAL AND METHODS

### 2.1 Data acquisition and processing

First, we used Broad GDAC Firehose website (http://gdac.broadinstitute.org/) to download the LGG genomic and clinical data, which is from the TCGA dataset. Second, we utilized RNASeqV2 normalized counts to calculate gene expression.^21^ Third, we used the GISTIC2.0 algorithm to calculate copy number alteration by the genome‐wide SNP6 array platform.^22^ At the same time, we used the JHU‐USC‐Human Methylation 450 platform output values to extract the beta values, which is calculated as (M/(M+U)). If there are multiple CpG islands for a single gene, we used the average beta values as the total methylation values.^23^ Finally, we summarized 34 core autophagic genes, all of which have been reported to participate in different important autophagy signalling pathways.^24^

### 2.2 Similarity network fusion and spectral clustering by multi‐omics profiles of autophagic genes

The gene expression, copy number alteration and methylation of 34 core genes of autophagy were aggregated using the SNF method, and the optimal number of clusters for spectral clustering was determined through the built‐in eigengap of SNF packages (Wang et al., 2018). SNF uses the affinity Matrix function to convert the distance matrix into a similarity matrix. In the distance matrix, the value larger, the distance farther; in the similarity matrix, the value larger, the matrix more similar. The input of affinity Matrix is three parameters: an existing distance matrix, parameters K and sigma. K is the number of neighbours, where the affinities of the neighbouring exterior are set to zero, and the affinities of the interior are normalized. Sigma is a hyperparameter of the scaling index similar kernel used to perform actual affinity calculations.^25^ SNF calculates and integrates similar networks from each data type of the patient. The purpose is to use the complementarity in the data to enhance the correlation between samples and facilitate the classification of samples. Through the SNF method, we obtained the patient similarity network and divided the patients into two subgroups.^26^

### 2.3 Identification of copy number and DNA methylation alterations related to SNF clustering subgroups

Subsequently, we analysed the copy number alteration and DNA methylation alterations for the two subgroups obtained by SNF clustering, and used Fisher's exact test between the samples in one SNF cluster subset and the other subset.^27^ Then, we obtained the statistical significance of the differences between frequencies of amplifications and deletions or differences between hypermethylation (the upper 33% of the DNA methylation level) and hypomethylation (the lower 33% of the DNA methylation level), and the P‐values were corrected by the Benjamini‐Hochberg (BH) method.^28^

### 2.4 Least absolute shrinkage and selection operator

The Lasso (Least absolute shrinkage and selection operator) method is a compression estimation method with the idea of reducing the variable set (order reduction). By constructing a penalty function, it can compress the coefficients of variables and make some regression coefficients become 0, and use the control parameter lambda for variable screening and complexity adjustment.^29^ We use the R package glmnet to perform lasso regression and analyse the data by fitting a generalized linear model.^30^

### 2.5 Multi‐omics‐based integrated autophagic network construction

Through the data preprocessing in 2.1, we obtained 14,909 genes with gene expression alterations, copy number alteration and DNA methylation alterations at the same time. To increase the precision of the calculation, we filtered the number of input genes. For the expression changed genes, we performed the Gene Ontology annotation, and got 297 genes which regulate autophagy^31^; at the same time, at least 20 related genes with copy number alteration or DNA methylation alterations are retained. Then, we calculated the Pearson correlation coefficients between the gene expression data and the corresponding copy number alteration data / DNA methylation data, and corrected the *p*‐values by the BH method (FDR < 0.05).^28^, ^32^ The genes whose Pearson correlation coefficient is greater than 0.5 or less than −0.5 are retained, and we obtained the positive / negative correlation between gene expression alterations and copy number alteration / DNA methylation. Finally, through the lasso method, the core autophagy network of the LGG datasets was constructed. The colour of nodes represents the calculation result of mRNA expression, copy number alteration, and DNA methylation. The arrow indicates the direction of assumed adjustment of the characteristic through the response variable; according to the positive or negative value of Pearson, the arrow shape is used to show up‐ or downregulation.

### 2.6 Cell culture

SW1088 cell line was purchased from ATCC (Manassas, VA, United States) and was cultured in a complete growth media [L‐15 Medium (Modified) Leibovitz (ATCC, VA, United States)] supplemented with 100 U/ml penicillin, 100 μg/ml streptomycin,10% FBS and 0.03% L‐glutamine. The cells were cultured in T75 ml flasks (Greiner Cellstar, VWR, PA, United States) and maintained at 37 degrees in an incubator. Cells were subcultured every 4 days, or when they reach 90% confluency. Passages 4–6 were used for all our experiments.

### 2.7 RNA knockdown or cDNA overexpression

The SW1088 cells were tested for mycoplasma contamination, and no contamination was found. Two siRNA for ZFP36L2 and RAB13 and two cDNAs for ZFP36L2 and RAB13 were obtained from GENECHEM. RT‐qPCR detects RNAi gene knockout and cDNA overexpression efficiency. SW1088 cells were transfected with two siRNA for ZFP36L2 and RAB13 and two cDNAs for ZFP36L2 and RAB13 and negative control siRNAs using Lipo3000 reagent (Invitrogen) according to the manufacturer's instructions. After transfection 24 h, the transfected cells were used for subsequent experiments. Total RNA was extracted from tissue or cultured cell with TRIZol (Invitrogen) following manufacturer s protocol. One microgram of total RNA was used for reverse transcription with TransScript All‐in One First‐Strand cDNA Synthesis SuperMix for RT‐qPCR (Transgene, AT341) to generate cDNA. And then real‐time fluorescent quantitative PCR was performed via iQ SYBR Green Supermix (Bio‐Rad) kits. The sequences of the primers for each gene detected are listed in Table 1.

**TABLE 1:** Sequences of primers used for RT‐RCR analysis (*Human*)

Primer Name	Forward sequence (5′−3′)	Reverse sequence (5′−3′)
ZFP36L2	CAACTCCACGCGCTACAAGA	CACTTTTCGCCGTACTTGCAC
RAB13	GATCCGCACTGTGGATATAGAGG	CCACGGTAGTAGGCAGTAGTTAT
ATG2B	TCCTTCAGGAAGAACAAAGCA	AAGCCTTACACGTGTGTCCA
FIP200	GGAACAACAGACCAATTTTAACAC	GCTCGATCTTCAGAAAGTGACTC
ULK1	TCGAGTTCTCCCGCAAGG	CGTCTGAGACTTGGCGAGGT
ATG16L1	CAGAGCAGCTACTAAGCGACT	AAAAGGGGAGATTCGGACAGA

### 2.8 Western blot

The expression levels of RAB13, ZFP36L2, ATG2B, LC3, Beclin‐1, ULK1, FIP200, SQSTM1/p62 and ATG16L1 proteins in SW1088 cells were evaluated using Western blot analysis. Approximately, SW108 cells were homogenized by using a polytron homogenizer in RIPA buffer with protein phosphatase inhibitor. After the lysates were harvested, protein concentrations of samples were measured using a bovine serum albumin kit (Bio‐Rad Laboratories, Hercules, USA). Analysis of Western blot was carried out as follows: equal amounts of protein were boiled for 10 min and separated by SDS‐PAGE on 8% 12% gels. The proteins were transferred to membranes and then incubated with primary antibodies: RAB13 (Abcam, ab180936), ZFP36L2 (Abcam, ab70775), ATG2B (Abcam, ab226832), LC3 (Abcam, ab51520), Beclin‐1 (Abcam, ab207612), ULK1 (Abcam, ab167139), FIP200 (CST, 12436s), SQSTM1/p62 (CST, 8025s), ATG16L1 (CST, 8089s) and β‐actin (Proteintech, IL, USA; 66009–1‐Ig). In addition, the secondary infrared antibodies goat anti‐rabbit IgG (Cell Signaling Technology #4410, 1:5,000) and goat anti‐mouse IgG (Cell Signaling Technology #4414, 1:5,000) were added.

### 2.9 Immunofluorescence

SW1088 cells were seeded onto the glass cover slips in 24‐well plates. After treatment, cells were fixed with 4% paraformaldehyde in PBS for 30 min. The slides were then washed three times with PBS and incubated with 0.2% Triton X‐100 (Sigma‐Aldrich, 9002–93–1) and 5% goat serum (Sigma‐Aldrich, G9023) for 30 min. Cells were incubated with indicated primary antibody overnight at 4°C and subsequently incubated with secondary antibody (TRITC, ab6718; FITC, ab6717) at room temperature for 1 h. Nuclei were finally stained with DAPI for 5 min. Images were captured using a confocal laser canning microscopy (Zeiss).

### 2.10 GFP/mRFP—LC3 transfection

The SW1088 cells were seeded into a 24‐well culture plate (2.0 × 10^4^ cells/well). After 24 h of incubation, the cells were transfected with GFP/mRFP‐LC3 (HB‐AP2100001, Hanbio, China) for 2 h. The transfected cells were then used in subsequent experiments for 48 h and analysed under a fluorescence microscope.

### 2.11 TCM database library preparing

The library for virtual screening was achieved from Traditional Chinese Medicine Database@ Taiwan (http://tcm.cmu.edu.tw/), the Encyclopedia of Traditional Chinese Medicine (http://www.tcmip.cn/ETCM/), Traditional Chinese Medicine Integrated Database (http://119.3.41.228:8000/tcmid/) and Yet another Traditional Chinese Medicine database (http://cadd.pharmacy.nankai.edu.cn/yatcm/home).

### 2.12 Homology modelling and molecular docking

The three‐dimensional structure of RAB13 was built using the SWISS‐MODEL server with an X‐ray crystal structure of Rab8A as the template (PDB ID: 6RIR). Molecular docking of compound libraries with RAB13 was performed using Accelrys Discovery Studio version 3.5 (Accelrys Inc.), and we used LibDock and CDOCK to dock the pre‐generated conformations into RAB13, by which ligands were able to be flexible corresponding to a rigid receptor with reasonable orientations. Finally, small‐molecule compounds with high scores were selected to further confirm their anti‐proliferative activities.

### 2.13 Annexin‐V/PI staining

The SW1088 cells were seeded into 6‐well culture plates with different concentrations of gallic acid and cultured for 24 h. The collected cells were then fixed with 500 ml PBS. The apoptotic ratio was measured by flow cytometry (Becton Dickinson, Franklin Lakes, NJ) after employing an Annexin‐V‐FLUOS Staining Kit (Roche).

### 2.14 Statistical analysis

We used the Wilcoxon rank sum test to identify significantly differentially expressed genes between the SNF clustering subgroups.^33^ All the experiments were performed independently at least three times, *p *< 0.05 was considered statistically significant. Differentially expressed genes were defined by median fold change >1.2 or <0.8. The upper 33% and lower 33% of gene expression were defined as high and low expression. We used GraphPad Prism 7.0 to make Kaplan‐Meier survival analysis,which is used to evaluate the association of gene expression with the OS and PFS of patients.^34^, ^35^ All statistical analyses were calculated by one‐way analysis of variance (ANOVA) followed by Scheffe's post hoc test. Data were obtained from repeated experiments and presented as mean ± SD. *p* < 0.05 was considered to have a statistical difference.

## 3 RESULTS

### 3.1 Multi‐omics approaches identify candidate autophagic regulators in LGG

**FIGURE 1:**
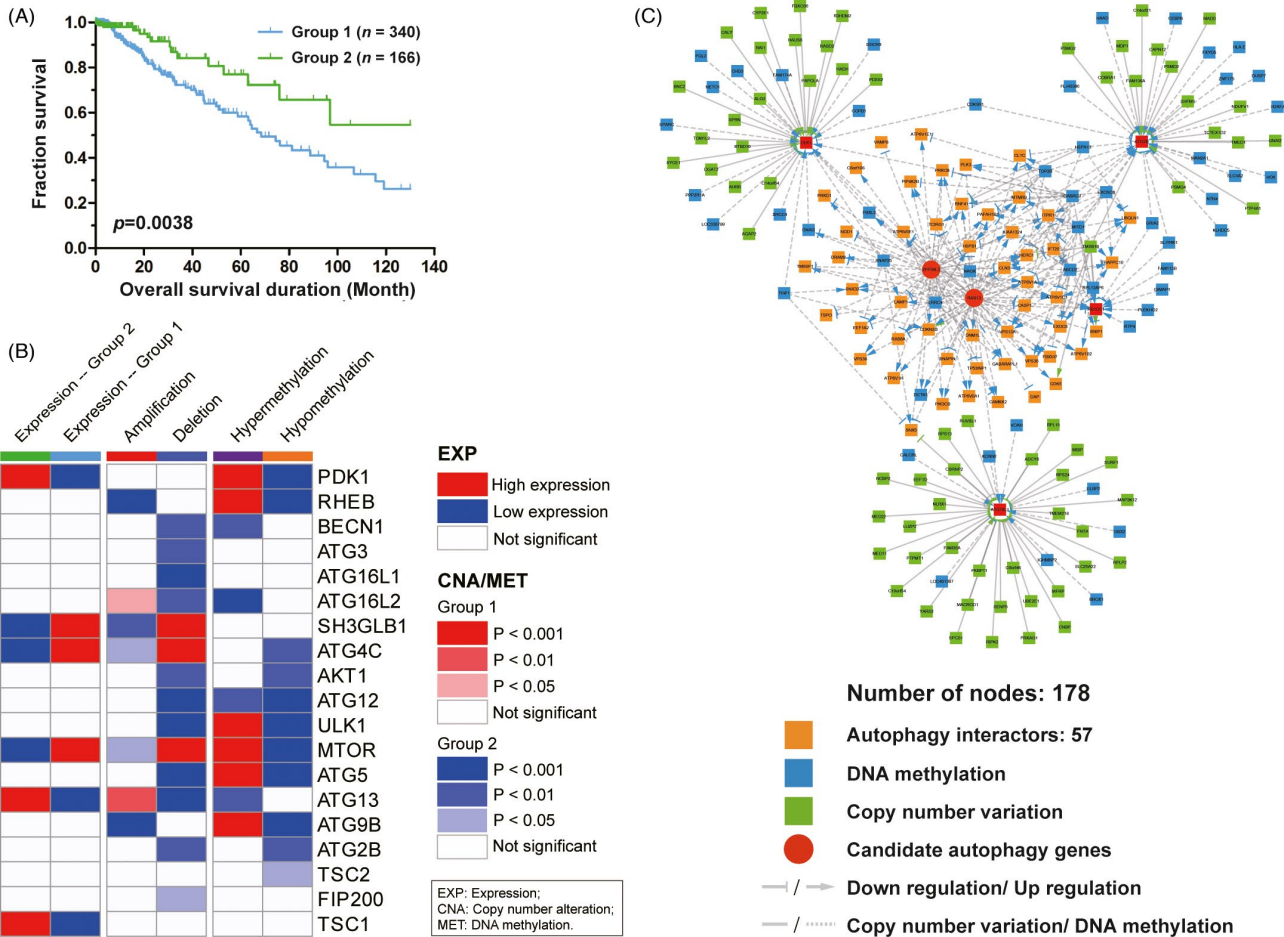
Integrated multi‐omics approaches to Identify of Candidate Autophagic Regulators in LGG (A) Progression‐free survival (PFS) analysis for the two autophagic subgroups, green represents group 1 and blue represents group 2. (B) Summary of the significant alterations of MET, CNA and EXP of the core autophagy genes for the two autophagic subgroups, *p* < 0.05 is regarded as alterations significantly. (C)The core autophagy network of the LGG datasets. There are 178 nodes in the core autophagy network, of which contains 57 autophagy interactors. Moreover, we use blue to indicate methylation alterations and green to indicate copy number alterations

We integrated gene expression, copy number alteration and methylation of 34 core autophagic genes (Table S1) to identify distinct the autophagic subgroups, and separated LGG patients into 2 best clusters (Figure S1A). Progression‐free survival (PFS) analysis for the two autophagic subgroups revealed that LGG patients in group 1 had significantly poorer PFS than those in group 2 (Figure 1A). Subsequently, we found that *SH3GLB1*, *ATG4C* and *MTOR* have higher expression levels in group 2, while *PDK1*, *ATG13* and *TSC1* have lower expression in group 2 (Figure 1B). Meanwhile, we noticed that *SH3GLB*, *ATG4C* and *MTOR* have amplification in group 2, while *ATG13* and *ATG16L2* have deletion in group 2 (Figure 1B). The differently expressed autophagic genes showed their significant differences in the frequencies of the copy number amplification or deletion and the frequencies of DNA hypermethylation or hypomethylation among the autophagic subgroups. Thus, we constructed a heatmap of core autophagic gene profiles, which provided an overview of the multi‐omics patterns of autophagy genes in the LGG subgroups (Figure S1B). *ATG13* had amplification and hypomethylation in group 2, with higher expression levels than group 1. At the same time, copy number deletion consistently induced lower mRNA expression of *MTOR*/*ATG4C* in group 2, which revealed the correlation between copy number alteration and gene expression was significant (Figure S1B). These results indicate that copy number alteration and methylation alterations mostly effect their gene expression. As mentioned above, we constructed the core autophagic network of the LGG datasets and found that all the copy number alteration and methylation alterations of autophagy core genes were shown to regulate their own expression (Figure S1C), which was consistent with some previous studies.^36^ Subsequently, we focused on the cross‐interactions of CNA‐EXP and MET‐EXP, and summarized 542 nodes and 1,101 interactions. Some positive autophagic regulators, such as *ULK1*, *FIP200*, *ATG16L1* and *ATG2B*, the number of interactions such as 39, 15, 44 and 40, could be regarded as hub genes. Then, we extracted the key autophagic process subnetwork from the autophagic network to identify the key autophagic genes that may play a critical role, which is contained 178 nodes and 337 interactions (Figure 1C). Among them, *ZFP36L2* and *RAB13* were found to be connected by abundant core autophagic genes and were regarded as candidate autophagic regulators. Hypermethylation of *ZFP36L2* could upregulate the expression of *ATG2B*, *FIP200* and *ULK1*. Similarly, hypermethylation of RAB13 could upregulate the expression of *ATG2B*, *ATG16L1*, *FIP200* and *ULK1*.

### 3.2 ZFP36L2 and RAB13 regulate the transcription of the key ATGs

**FIGURE 2:**
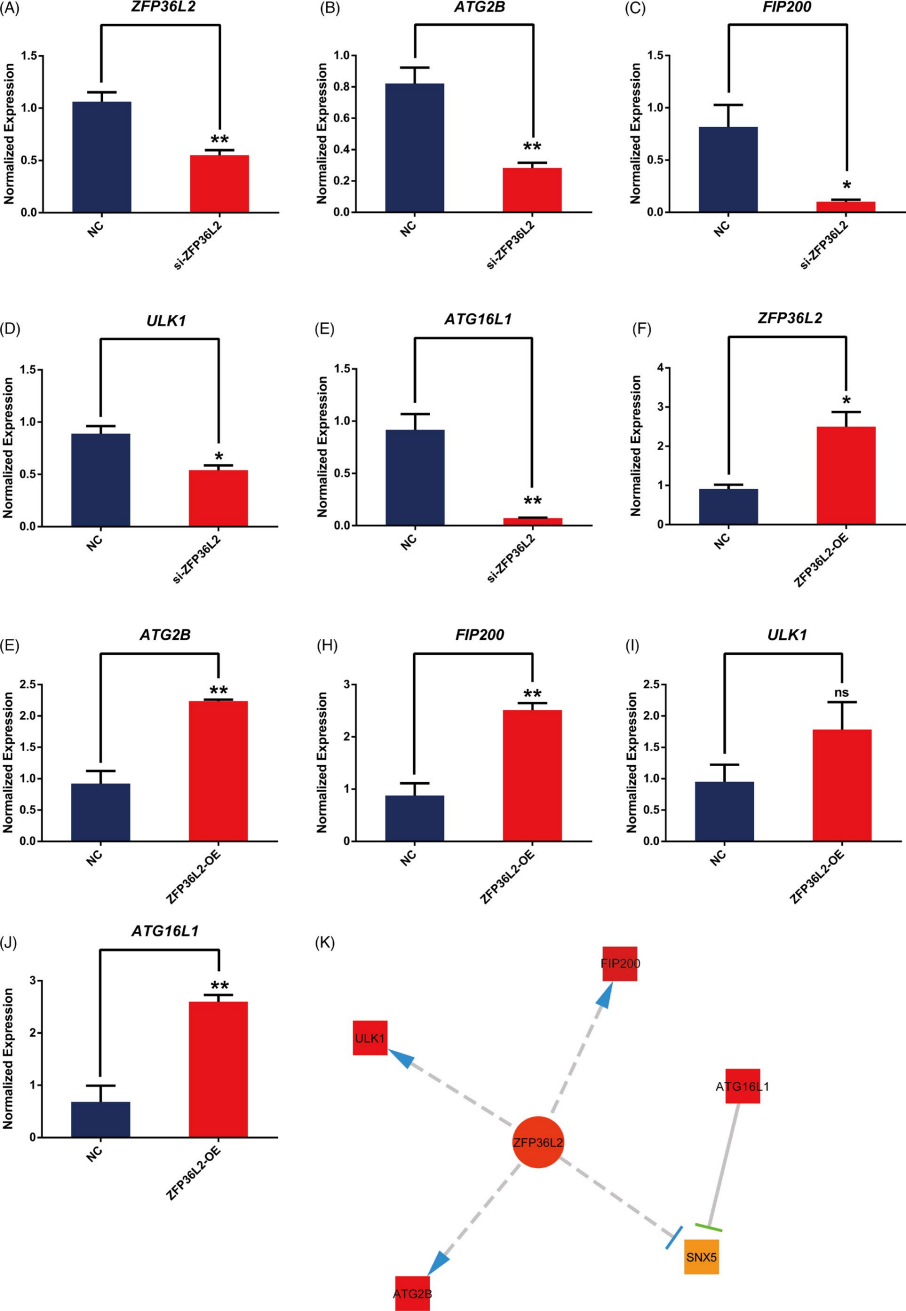
Effects of ZFP36L2 on autophagic gene mRNA expression. (A–E) The expression of ATG2B, FIP200, ULK1 and ATG16L1 in NC and si‐ZFP36L2 groups quantified by RT‐qPCR. All data were representative of at least three independent experiments. *p < 0.05, **p < 0.01, ***p < 0.001 vs the NC group. (F–J) The expression of ATG2B, FIP200, ULK1 and ATG16L1 in NC and ZFP36L2‐OE groups quantified by RT‐qPCR. All data were representative of at least three independent experiments. *p < 0.05, **p < 0.01, ***p < 0.001 versus the NC group. (K) Key autophagic genes related to ZFP36L2 analysed through the network

**FIGURE 3:**
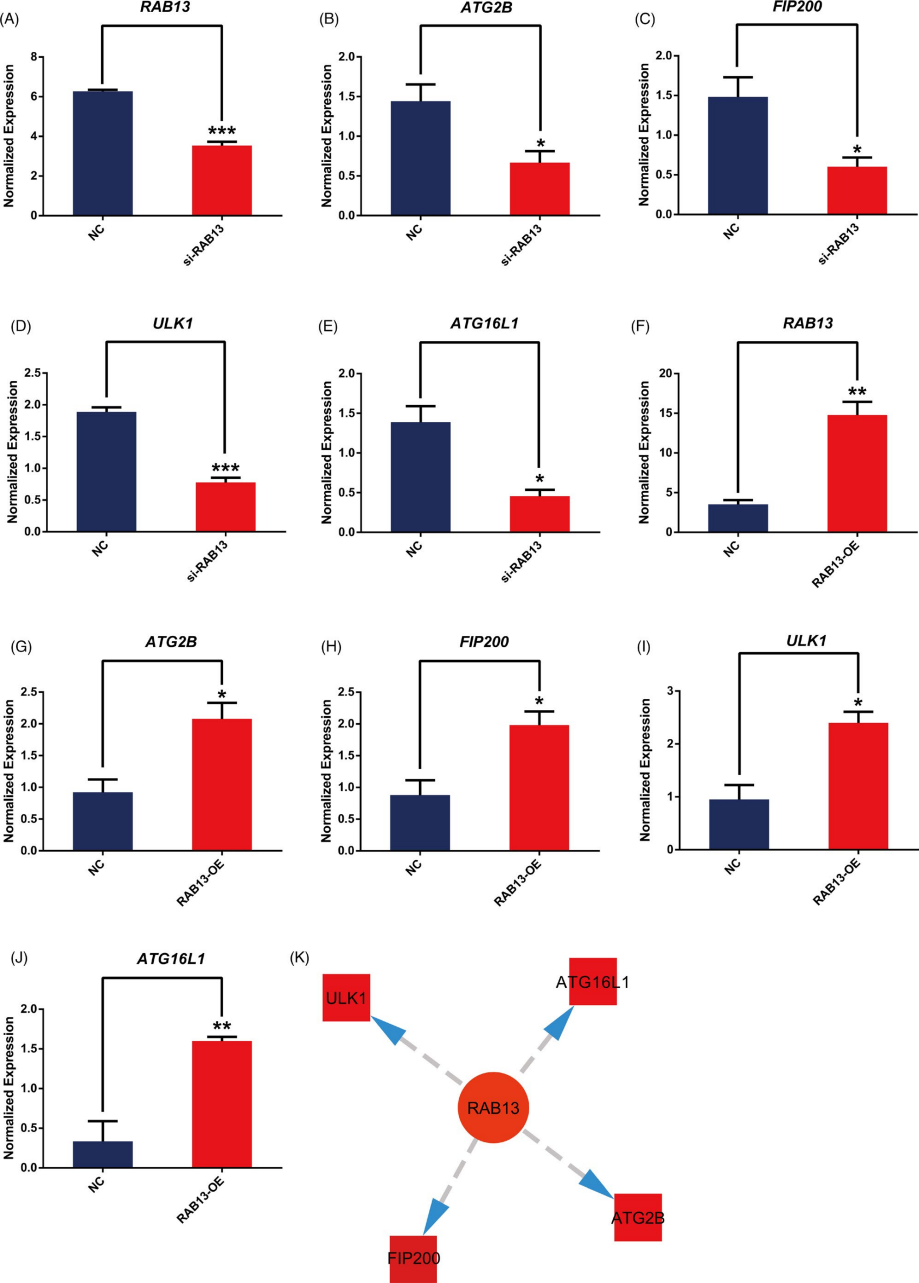
Effects of RAB13 on autophagic gene mRNA expression. (A–E) The expression of ATG2B, FIP200, ULK1and ATG16L1 in si‐NC and si‐RAB13 groups quantified by RT‐qPCR. All data were representative of at least three independent experiments. * *p* < 0.05, ** *p* < 0.01, *** *p* < 0.001 vs the NC group. (F–J) The expression of ATG2B, FIP200, ULK1 and ATG16L1 in vector and RAB13‐OE groups quantified by RT‐qPCR. All data were representative of at least three independent experiments. * *p* < 0.05, ** *p* < 0.01, *** *p* < 0.001 versus the NC group. (K) Key autophagic genes related to RAB13 analysed through the network

The results of multi‐omics network data indicated that ZFP36L2 and RAB13 can regulate the autophagy of LGG by regulating the expression of key autophagy genes such as ULK1, FIP200, ATG16L1 and ATG2B. Therefore, we verified the relationship between the regulators and above‐mentioned autophagy‐related genes by knockdown and overexpression ZFP36L2 and RAB13 in Human Brain Cancer Cell Lines SW1088 cells. The mRNA transcription levels of *ULK1*, *FIP200*, *ATG16L1* and *ATG2B* were down‐ regulated when *ZFP36L2* is knocked down (Figure 2A–E). Conversely, the overexpression of *ZFP36L2* enhanced their transcription levels (Figure 2F–J). Similarly, the knockdown or overexpression of RAB13 decreased or increased the transcription level of these genes respectively (Figure 3A–J). Taken together, the results suggest that both ZFP36L2 and RAB13 may be positive regulators of autophagy (Figures 2K and 3K).

### 3.3 ZFP36L2 and RAB13 are positive autophagic regulators

**FIGURE 4:**
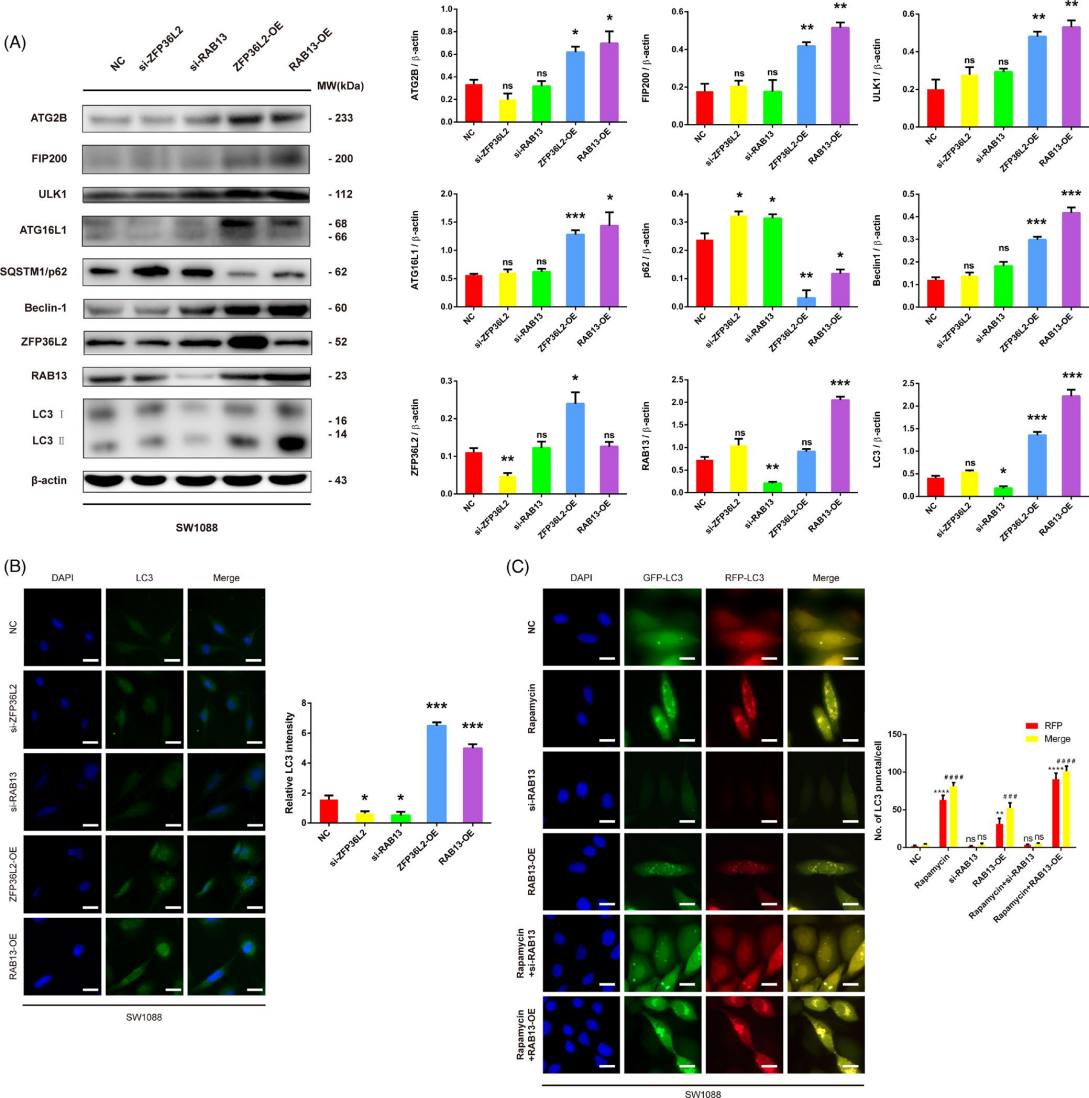
Effects of ZFP36L2 or RAB13 overexpression or knockdown on the expression of autophagic proteins in SW1088 cells; (A) SW1088 cells transfected with vector, si‐ZFP36L2, si‐RAB13, c‐ZFP36L2, c‐RAB13, autophagic proteins were quantified by Western blot. All data were representative of at least three independent experiments. ns, no significance; p* < 0.05, **p < 0.01, ***p < 0.001. (B) The expression and location of LC3 were detected by immunofluorescence. Scale bar = 20 µm. (C) After SW1088, cells were transfected with si‐RAB13 and c‐RAB13 for 24 h in the presence or absence of rapamycin (10 nM). Then, SW1088 cells was transfected with GFP/mRFP‐LC3 plasmid, representative images and quantitative analysis of LC3 puncta were shown. Scale bar = 20 µm. Merge compared with the NC group, # *p* < 0.05, ## *p* < 0.01, ### *p* < 0.001, ns, no significance; RFP compared with the NC group, *p < 0.05, ** *p* < 0.01, *** *p* < 0.001, ns, no significance

To verify the effect of ZFP36L2 and RAB13 on autophagy, we investigated the effect of ZFP36L2 and RAB13 on some autophagic biomarkers and related autophagic regulators. Overexpression of ZFP36L2 upregulated transformation from LC3Ⅰ to LC3Ⅱ, with a decrease of p62 (Figure 4A). Meanwhile, the expression of ATG2B, FIP200, ULK1, ATG16L1 and Beclin‐1 was upregulated following ZFP36L2 overexpression (Figure 4A). However, the transformation from LC3Ⅰ to LC3Ⅱ and the expression levels of ATG2B, FIP200, ULK1, ATG16L1 and Beclin‐1 were not changed significantly (*p *> 0.05) after ZFP36L2 knockdown, which may imply some unknown changes at the transcriptional level (Figure 4A). To further confirm the effect of ZFP36L2 knockdown on LC3 and autophagy, we used immunofluorescence to detect the expression of LC3 and found that ZFP36L2 knockdown caused decrease of LC3 fluorescence intensity. More importantly, ZFP36L2 overexpression led to a notable increase of LC3 fluorescence intensity (Figure 4B). The altered autophagic biomarkers and the results of immunofluorescence demonstrate that ZFP36L2 may act as a positive regulator of autophagy.

In addition, overexpression of RAB13 led to an increase of transformation from LC3Ⅰ to LC3Ⅱ, and a decrease of p62, the expression of ATG2B, FIP200, ULK1, ATG16L1 and Beclin‐1 were upregulated (Figure 4A). Different with the results of ZFP36L2, the knockout of RAB13 gene reduced the conversion of LC3Ⅰ to LC3Ⅱ and increased the protein expression level of p62, suggesting that inhibition of RAB13 may inhibit autophagy (Figure 4A). Additionally, consistent with the results of qPCR, no changes of these proteins were observed after RAB13 knockdown (Figure 4A). To explore the impact that RAB13 overexpression or knockdown had on autophagy, immunofluorescence was also applied to detect autophagy. LC3 fluorescence intensity of the stable RAB13 overexpression group was significantly higher than that of control group, while LC3 fluorescence intensity of RAB13 knockdown group reduced markedly (Figure 4B). Similarly, GFP/mRFP‐LC3 puncta of RAB13 overexpression group were significantly higher than control group, while GFP/mRFP‐LC3 puncta of RAB13 knockdown group were not changed significantly (Figure 4C). Interestingly, knockdown RAB13 markedly reduced the GFP/mRFP‐LC3 puncta that induced by rapamycin (Figure 4C). These results support the view that RAB13 gene overexpression activates complete autophagy flux in SW1088 cells. Taken together, RAB13 acts as a positive regulator in autophagy.

### 3.4 RAB13 is a potential druggable target in LGG

**FIGURE 5:**
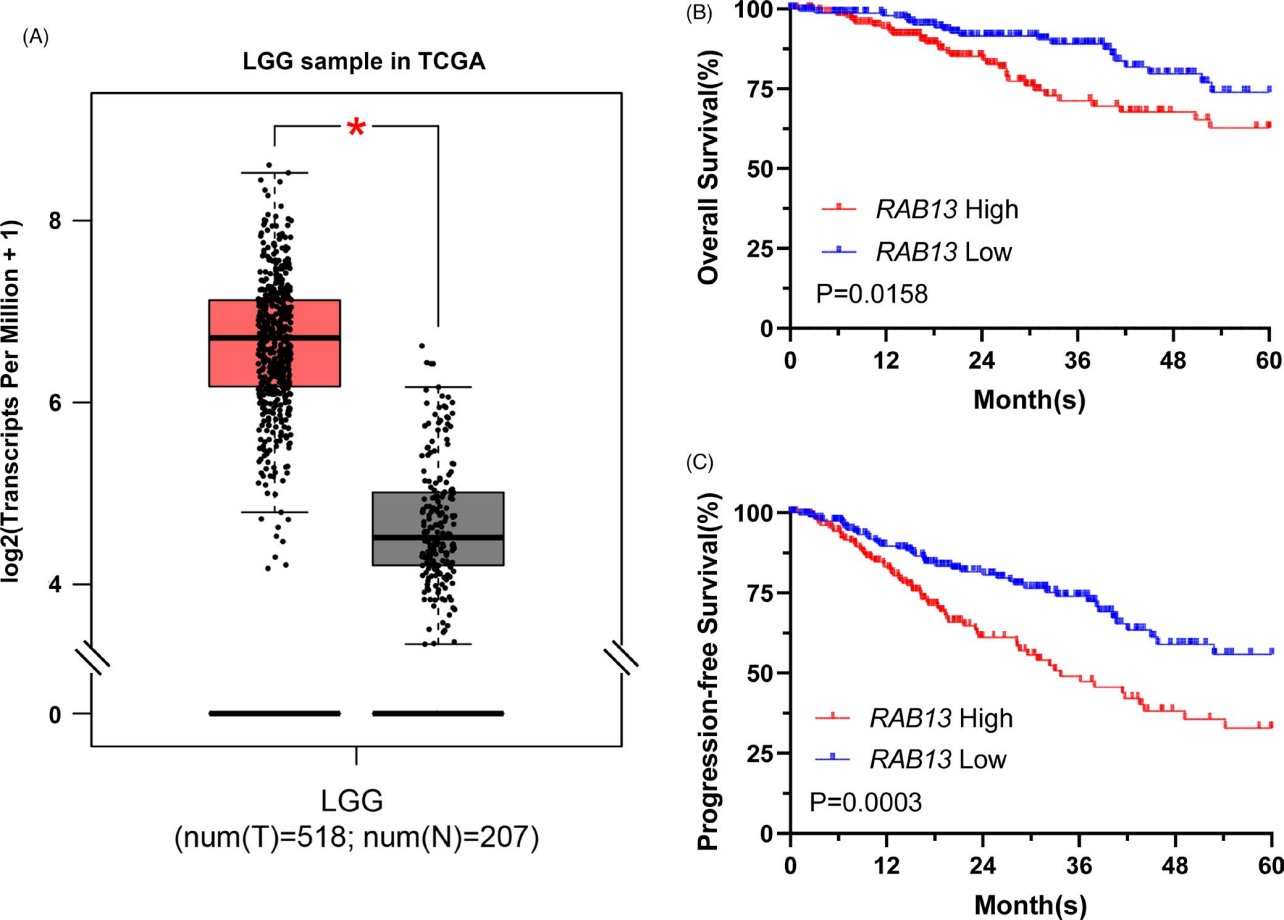
RAB13 are candidate prognostic biomarkers of LGG. (A) RAB13 expression is significantly higher in LGG tumours than in Normal. (B) and (C) Kaplan‐Meier curves of overall survival and progression‐free survival under high RAB13 expression and low RAB13 expression. The upper 33% and lower 33% of gene expression were considered high and low

Rab13, an Rab GTPases, plays a key role in the delivery of cargo to the plasma membrane, disruption of cell‐cell adhesions, as well as driving cell migration through rearrangement of the actin cytoskeleton and surface delivery of selective proteins. Rab13 is recognized as an important driver of cancer progression since the abnormal function of Rab13 is often observed in cancer.^37^ To explore the function of RAB13 in LGG, we investigated TCGA LGG gene expression difference between LGG and Normal tissue. We found that RAB13 expression was upregulated in LGG (Figure 5A). Moreover, higher expression of RAB13 were found to be closely associated with poor prognosis (Figure 5B, C). Together, these results indicate that inhibiting RAB13 may be a promising strategy for the LGG therapy.

### 3.5 Gallic acid negatively regulates autophagy by inhibiting RAB13 in LGG

**FIGURE 6:**
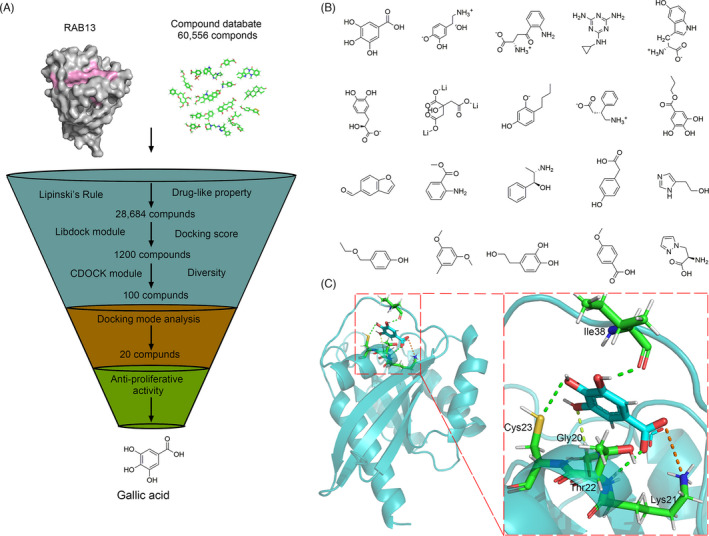
In silico high‐throughput screening of candidate small‐molecule compounds targeting RAB13. (A) Virtual screening protocol for the identification of the novel RAB13 inhibitor. (B) The top twenty candidate small‐molecule compounds targeting RAB13 from Traditional Chinese Medicine Database. (C) The interactions between Gallic acid and RAB13. A Zoom view shows that Gallic acid formed hydrophobic interactions with the binding pocket

**FIGURE 7:**
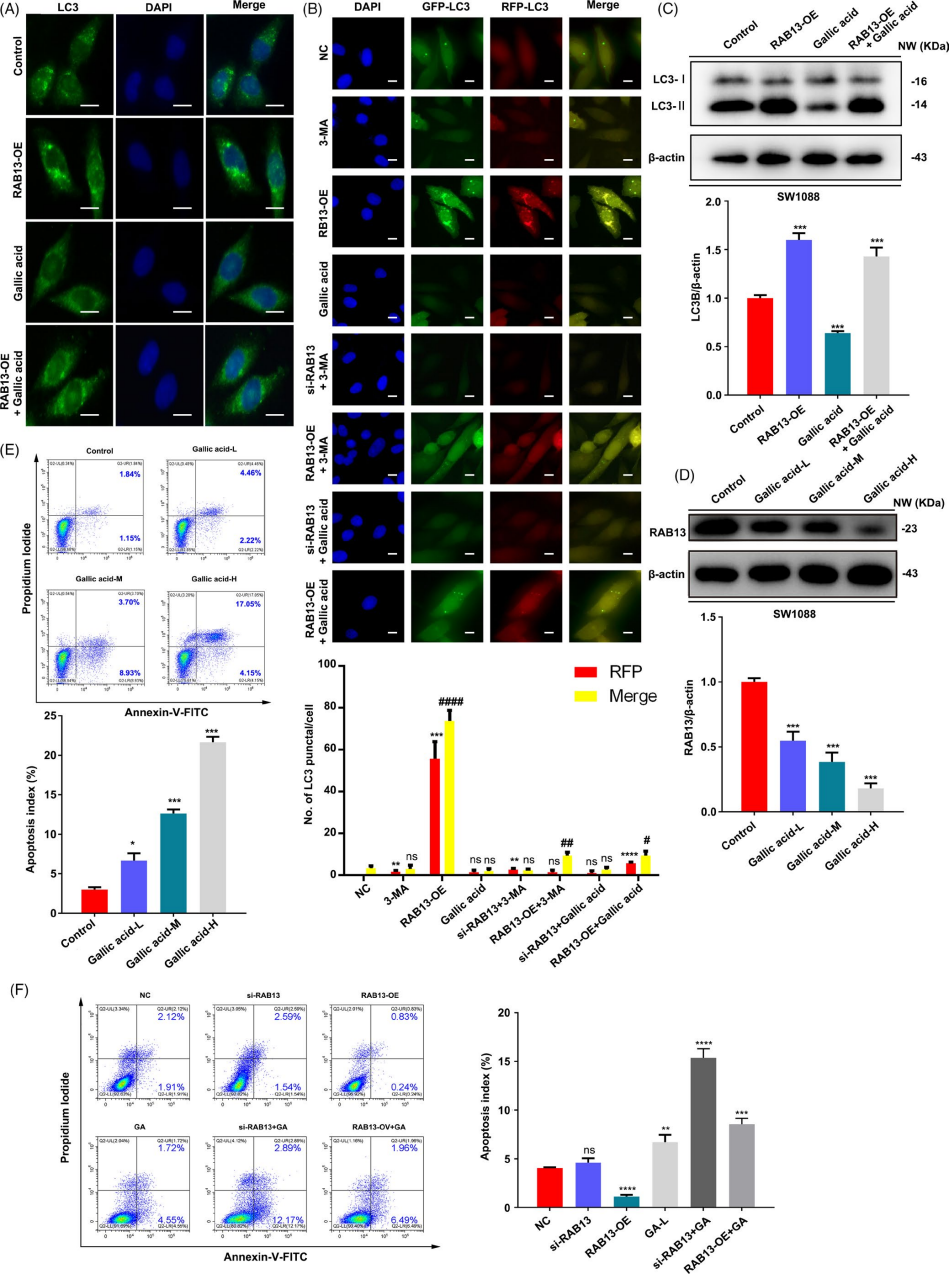
Gallic acid inhibits autophagy and induces cell death in SW1088 cells. (A) SW1088 cells were transfected with vector or c‐RAB13, and then treated with or without gallic acid. The expression and location of LC3 were detected by immunofluorescence. Scale bar = 20 µm. (B) After SW1088 cells were transfected with si‐RAB13 and c‐RAB13 for 24 h, after co‐incubation with gallic acid (1 mM) or 3‐MA (10 nM). Then, SW1088 cell was transfected with GFP/mRFP‐LC3 plasmid, representative images and quantitative analysis of LC3 puncta were shown. Scale bar = 20 µm. (C) SW1088 cells were transfected with vector or c‐RAB13, and then treated with or without Gallic acid for 24 h. The expression of LC3 was detected by Western blot analysis. All data were representative of at least three independent experiments. Merge compared with the NC group, # *p* < 0.05, ## *p* < 0.01, ### *p* < 0.001, ns, no significance; RFP compared with the NC group, *p < 0.05, ** *p* < 0.01, *** *p* < 0.001, ns, no significance. (D) SW1088 cells were treated with different concentrations of gallic acid for 24 h. The expression of RAB13 was detected by Western blot analysis. All data were representative of at least three independent experiments. GA‐L, Gallic acid 1 mM; GA‐M, Gallic acid 2 mM; GA‐H, Gallic acid 3 mM; ns, no significance; P*<0.05, **p < 0.01, ***p < 0.001. (E) SW1088 cells were treated with different concentrations of gallic acid for 24 h, and the apoptosis ratios were determined by flow cytometry analysis of Annexin‐V/PI double staining. GA‐L, Gallic acid 1 mM; GA‐L, Gallic acid 2 mM; GA‐L, Gallic acid 3 mM. (F) SW1088 cells were transfected with vector or c‐RAB13, and then treated with or without Gallic acid for 24 h, and the apoptosis ratios were determined by flow cytometry analysis of Annexin‐V/PI double staining

In this study, we built the three‐dimensional structure of RAB13 using the SWISS‐MODEL server with an X‐ray crystal structure of Rab8A as the template (PDB ID: 6RIR). Then, we utilized a multiple docking strategy to discover the lead inhibitor of RAB13 (Figure 6A). A total of 286,712 compounds in the Traditional Chinese Medicine (TCM) Databases were filtered by the Lipinski's rule of five, yielding 192,453 retained compounds. Subsequently, structure‐based molecular docking was performed using LibDock and CDOCKER, yielding 100 compounds with high docking score. We analysed the docking mode and selected 20 top hits compounds as candidate inhibitors (Figure 6B). Gallic acid exhibited the best score (CDOCKER energy 59.3629; CDOCKER interaction energy 57.8075) and it formed hydrophobic interactions with the binding pocket of RAB13 (Figure 6C). Thus, we examined the effect of gallic acid on SW1088 cells autophagy. Gallic acid was able to inhibit RAB13 in SW1088 cells, which alleviate the increase of LC3 intensity caused by overexpression of RAB13 (Figure 7A). In addition, the GFP/mRFP‐LC3 puncta induced by overexpression RAB13 were remarkably reversed when treatment with 3‐MA or gallic acid (Figure 7B). Moreover, the increase of LC3‐Ⅱ caused by overexpression of RAB13 was downregulated as demonstrated by Western blot (Figure 7C). Gallic acid can reduce the protein level of RAB13 in a dose‐dependent manner (Figure 7D). We found that gallic acid could significantly induce cell death in a dose‐dependent manner (Figure 7E). More importantly, when knockdown of RAB13 with siRNA, gallic acid can further significantly induce cell apoptosis, but overexpression of RAB13 not affect the apoptosis by gallic acid obviously as knockdown RAB13 (Figure 7F). Taken together, these results suggest that gallic acid can inhibit autophagy by targeting RAB13 and exert an anti‐tumour activity in LGG.

## 4 DISCUSSION

The protective autophagy promotes the initiation and progression of glioma and induces chemoresistance.^6^, ^38^, ^39^ However, the more detailed effect of autophagy on glioma remains to be clarified. Some prognostic predictor, such as GRID2, FOXO1, MYC, PTK6, IKBKE, BIRC5 and TP73, has been screened based on the analysis of autophagy‐related genes (ATGs) by a novel prognostic model and nomogram.^40^ In addition, several researches identified autophagic biomarkers of LGG according the data of TCGA and the Chinese Glioma Genome Atlas (CGGA).^41^, ^42^, ^43^ However, these current studies may lead to incomplete exploitation of the cancer genome because of one‐dimensional analyses of somatic mutations, and all these studies as mentioned above only conducted bioinformatics analysis, lacking validations of these identified targets.

In this study, we analysed a variety of omics data from LGG, including EXP, MET, and CNA data, with the SNF method and the LASSO algorithm, and finally discovered two LGG candidate positive autophagy modulators, ZFP36L2 and RAB13, which are closely related to the core autophagy regulators, ULK1, FIP200, ATG16L1 and ATG2B. Subsequently, we found that ZFP36L2 and RAB13 had a regulatory relationship with the four core autophagy regulators ULK1, FIP200, ATG16L1 and ATG2B. Both transcription level and protein expression level of the four autophagy regulators and LC3 puncta were increased by ZFP36L2 and RAB13 overexpression, suggesting ZFP36L2 and RAB13 may be positive regulators of autophagy.

ZFP36L2 has been reported to be associated with tumour invasion. As a latest example, mutations in ZFP36L2 were found enriched in metastatic CRC and associated with poor overall survival. Knockout of ZFP36L2 with CRIPSR/Cas 9 facilitates the metastatic potential of CRC cells, illuminating ZFP36L2 may act as a suppressor in cancer progression.^21^ However, the rational drug design to selectively target ZFP36L2 requires the complete structural analysis of ZFP36L2.

Autophagy involves dynamic crosstalk with different stages of intracellular vesicle trafficking, where Golgi‐associated Rab GTPases function as important mediators.^44^, ^45^ Eight Golgi‐associated Rab GTPases, Rab1, Rab6, Rab9, Rab11, Rab24, Rab30, Rab33 and Rab37 were found to participate in the initiation, formation, maturation and fusion of autophagosome and played crucial role in autophagy.^44^, ^46^ Upregulation of the active form of Rab13 promotes autophagy through interaction with Grb2 in vascular endothelial cells.^47^ In this study, we found that RAB13 participates in autophagy through ATG2B, FIP200, ULK1, ATG16L1 and Beclin‐1, indicating its important biological function in LGG autophagy.

TCM is considered as good sources of anti‐tumour drug candidates since most of them are multi‐target and low toxic. More importantly, most TCM possess good ability to penetrate blood‐brain barrier, which is particularly benefit in treating gliomas.^48^ To date, a large number of TCM databases have been established,^49^, ^50^, ^51^ providing extensive natural‐compound candidates for the discovery of novel drugs. Recently, TCM cyclovirobuxine D, derived from Buxus microphylla, was reported to possess therapeutic potential on LGG through blocking cell cycle and activating apoptosis. However, the more detailed mechanism of cyclovirobuxine D to treat LGG has not been investigated.^52^ In our study, we screened multi‐TCM databases and identified gallic acid as a novel potential RAB13 inhibitor, which was confirmed to negatively regulate autophagy as well as to induce cell death in SW1088 cells. Our study offers a new candidate TCM compound, gallic acid, to treat LGG with detailed mechanism—by inhibiting RAB13 and suppressing autophagy.

Nevertheless, recent studies also indicated that gallic acid activates autophagy by inhibiting ULK1 phosphorylation, thereby suppresses cardiac hypertrophic remodelling and heart failure.^53^ Terminalia bellirica (TB) extract could induce anti‐cancer activity by activating apoptosis and autophagy in oral squamous cell carcinoma (OSCC). The results also showed that TB extract containing gallic acid attenuated autophagosomal lysosomal fusion in OSCC cell line Cal33 without changing lysosomal activity. It was speculated that the presence of gallic acid caused the damage of autophagosomal lysosomal fusion.^54^ Another study also showed that the specific anti‐cancer mechanism of gallic acid in OSCC, it was found that OSCC cells treated with gallic acid and gamma‐irradiated gallic acid (GAIR) could initiate autophagy in the early stage. Finally, enhanced p62 expression and constant LAMP1 expression suggested that the autophagy flux was inhibited without changing the solute membrane protein. GAIR inhibit autophagy and regulate OSCC cell death. Moreover, gallic acid shows a stronger synergistic anti‐cancer effect when combined with autophagy inhibitors CQ and Bafilomycin A1.^55^ In conclusion, similar experimental results suggest that gallic acid can inhibit autophagy flux by inhibiting autophagy lysosomal fusion, and finally lead to OSCC cell death. Our study shows that gallic acid could target RAB13 in LGG. This result provides new clues to reveal the RAB13 participate in autophagy process. In short, the extensive and in‐depth mechanism of GA regulating autophagy may need further research.

In summary, based upon multi‐omics approaches, we finally identified two new autophagic regulators, ZFP36L2 and RAB13, as potential druggable targets in LGG. Moreover, we showed that gallic acid were able to inhibit autophagy by target RAB13 and induce cell death in LGG. Thus, these findings not only demonstrate the key autophagic regulators ZFP36L2 and RAB13 as potential druggable targets in LGG, but also provide gallic acid as small‐molecule inhibitor of autophagy from TCM for future cancer drug development.

## CONFLICT OF INTEREST

The authors declare no conflict of interest.

## ACKNOWLEDGEMENT

This work was supported by grants from National Natural Science Foundation of China (Grant No. 82172649), The Research Foundation for Administration of traditional Chinese Medicine of Sichuan Province (Grant No. 2020HJZX002), and Key R&D Program of Sichuan Province (Grant No. 2021YFS0046).

## AUTHOR CONTRIBUTIONS

Y.W.Z, L.H. and B.L. directed the project and wrote the manuscript. W.S. and M.R.L. performed the experiments, assisted with project management and edited the revised manuscript. H.D.T., Y.M.C., W.K.J., S.O.Z. and R.Y. Z. performed omics data and assisted with writing the manuscript. All authors provided intellectual input into the study. All authors read and approved to submit the final manuscript.

## Supporting information

Fig S1

Table S1

## Data Availability

The data that support the findings of this study are available from the corresponding author upon reasonable request.
