# Supplementary material for: Identification of autophagic target RAB13 with small‐molecule inhibitor in low‐grade glioma via integrated multi‐omics approaches coupled with virtual screening of traditional Chinese medicine databases
Source: Cell Prolif. 2021 Oct 10;54(12):e13135. doi: 10.1111/cpr.13135 (PMC8666277; doi:10.1111/cpr.13135)
Supplement: Supplementary file 1 — Fig S1 [file CPR-54-e13135-s002.docx]

**Supplementary Materials**

**Integrated Multi-omics Approaches and** **Traditional Chinese Medicine Databases Identify Autophagic Druggable Target RAB13 with Small-Molecule Inhibitor in Low-grade Glioma**

Wei Su^#^, Minru Liao^#^, Huidan Tan^#^, Yanmei Chen, Rongyan Zhao, Wenke Jin, Yiwen Zhang*, Li He*, Bo Liu*

Department of Neurology, State Key Laboratory of Biotherapy and Cancer Center, West China Hospital of Sichuan University, Chengdu 610041, China

Supplementary Figure S1


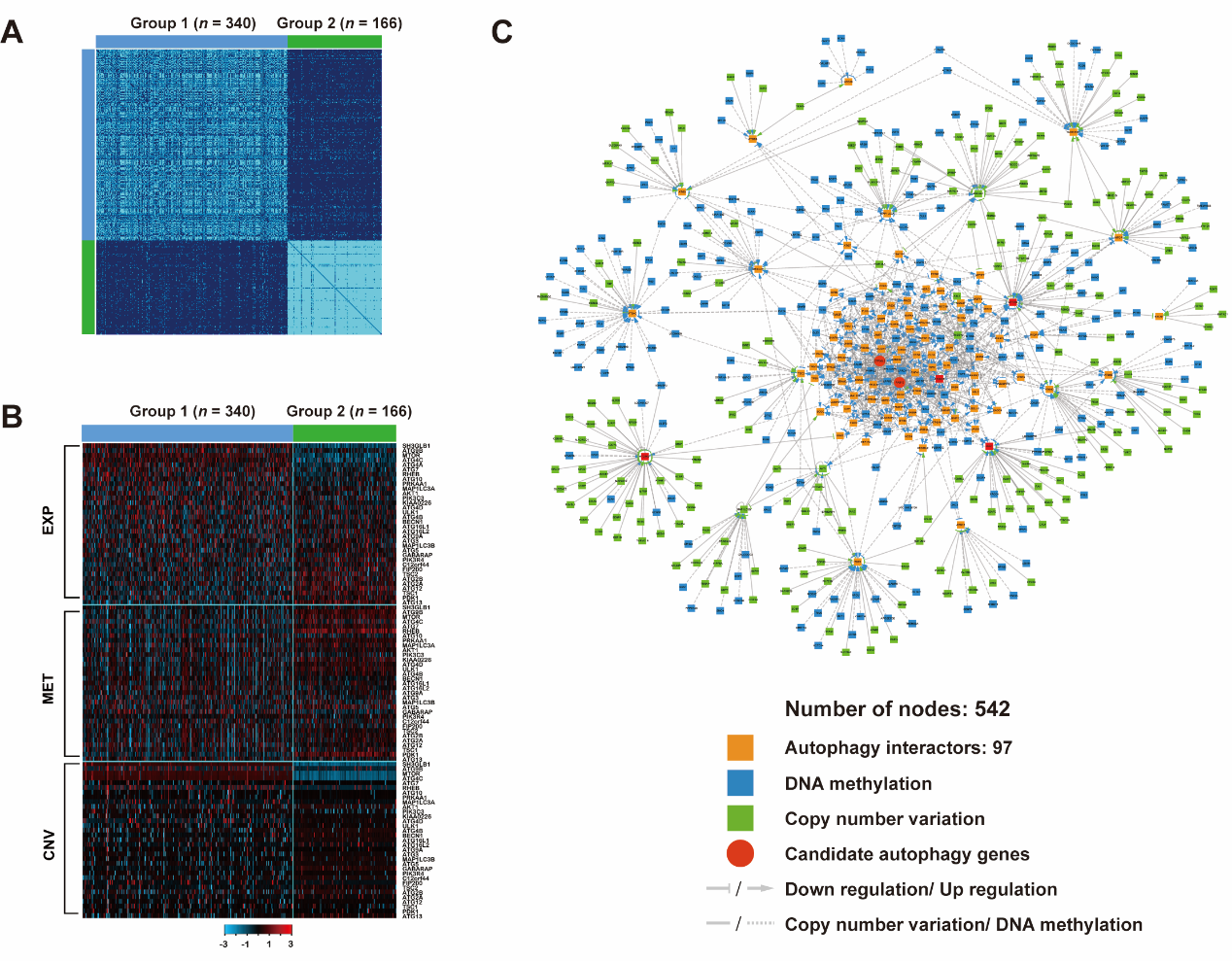


**Supplementary Figure** **|**1 Integrated multi-omics approaches to Identify of Candidate Autophagic Regulators in LGG (**A**)Patient similarity matrix of two autophagic subgroups of LGG patients. (**B**) A heatmap of core autophagic gene profiles, which provided an overview of the multi-omics patterns of autophagy genes in the LGG subgroups. (**C**) The color of nodes represents the calculation result of mRNA expression, copy number alteration, and DNA methylation. The arrow indicates the direction of assumed adjustment of the characteristic through the response variable; according to the positive or negative value of Pearson, the arrow shape is used to show up or down regulation.
